# The genome sequence of the Black Lace-weaver spider, *Amaurobius ferox (*Walckenaer, 1830)

**DOI:** 10.12688/wellcomeopenres.21080.1

**Published:** 2024-03-01

**Authors:** Sérgio Henriques, Natural History Museum Genome Acquisition Lab, Darwin Tree of Life Barcoding collective, Wellcome Sanger Institute Tree of Life Management, Samples and Laboratory team, Wellcome Sanger Institute Scientific Operations: Sequencing Operations, Wellcome Sanger Institute Tree of Life Core Informatics team, Tree of Life Core Informatics collective, Darwin Tree of Life Consortium

**Affiliations:** 1 Indianapolis Zoological Society, Indianapolis, Indiana, USA

**Keywords:** Amaurobius ferox, Black Lace-weaver, genome sequence, chromosomal, Araneae

## Abstract

We present a genome assembly from an individual female *Amaurobius ferox* (the Black Lace-weaver; Arthropoda; Arachnida; Araneae; Amaurobiidae). The genome sequence is 3,564.8 megabases in span. Most of the assembly is scaffolded into 23 chromosomal pseudomolecules, including the X _1_, X _2_ and X _3_ sex chromosomes. The mitochondrial genome has also been assembled and is 14.24 kilobases in length.

## Species taxonomy

Eukaryota; Opisthokonta; Metazoa; Eumetazoa; Bilateria; Protostomia; Ecdysozoa; Panarthropoda; Arthropoda; Chelicerata; Arachnida; Araneae; Araneomorphae; Entelegynae; RTA clade; Amaurobiidae; *Amaurobius*; *Amaurobius ferox* (Walckenaer, 1830) (NCBI:txid336583).

## Background

*Amaurobius ferox* (Walckenaer, 1830), commonly known as the Black Lace-weaver, is a species of the Amaurobiidae family. It is one of three *Amaurobius* species occurring in Britain, which share a characteristic lace-like web ( Miller *et al.*, 2010; Řezáč *et al.*, 2017). When viewed dorsally, *Amaurobius ferox* is very similar to the other two British species of this genus, *Amaurobius fenestralis* and *Amaurobius similis*, with markings in females ranging from dusky to ill-defined ( Roberts, 1995). All three species have rather wide distributions in Britain, and are found in diverse habitats. There are some differences among them in terms of their micro-habitat preferences. *Amaurobius fenestralis* prefers to build its webs in woodlands, usually at the soil level (in the leaf litter or under fallen logs and large stones), although it can also be found in crevices of tree bark or in plants with stiff structure and dense foliage (such as hedgerows). *Amaurobius similis* is perhaps the most familiar species of this genus as it is often found in houses and sheds (even in very urban settings), building its webs in crevices of walls and windows, although it can also share some of the same woodland habitats as *A. fenestralis*, especially near habitations. *Amaurobius ferox* prefers to build its webs under stones or rubble and seems to prefer shadier, more humid microhabitats in walls or in tunnels or caves entrances ( Bee *et al.*, 2017; Bristowe, 1958; Roberts, 1995). Despite these differences, *Amaurobius* species are adaptable, and so the location of the web is not a reliable way to distinguish them ( Bee *et al.*, 2017; Roberts, 1995). Males of all three species emerge in the same seasons, mostly late summer and autumn, with some males being found in late winter and spring. However, *Amaurobius ferox* appears to have its male peak emergence season in spring. Phenology is thus also unreliable for distinguishing this species, and analysis of the genitalia is strongly encouraged for accurate separation of the species ( Bee *et al.*, 2017).

*Amaurobius ferox* males have a distinctive pedipalp with two tibial apophysis (one being wide and blunt, the other hook-shaped), females have a distinct epigyne, with a triangular shape where the base is curved inwards and the tip points towards the spinnerets ( Roberts, 1995). Maternal care in this sub-social species is highly complex, where the mother produces trophic eggs for her spiderlings ( Kim & Roland, 2000), with spiderlings and their mother practising matriphagy ( Kim & Horel, 1998). The young sub-social spiders also cooperate in prey capture ( Kim *et al.*, 2005), and synchronise their development ( Kim, 2001).

Given the intricate reproductive behaviours of *A. ferox*, decoding its genome offers valuable insights into the genetic underpinnings of these complex patterns. In this study, we present a chromosomally complete genome sequence for *Amaurobius ferox*, derived from one female specimen collected at Chobham Common, Surrey, UK.

## Genome sequence report

**Figure 1.**
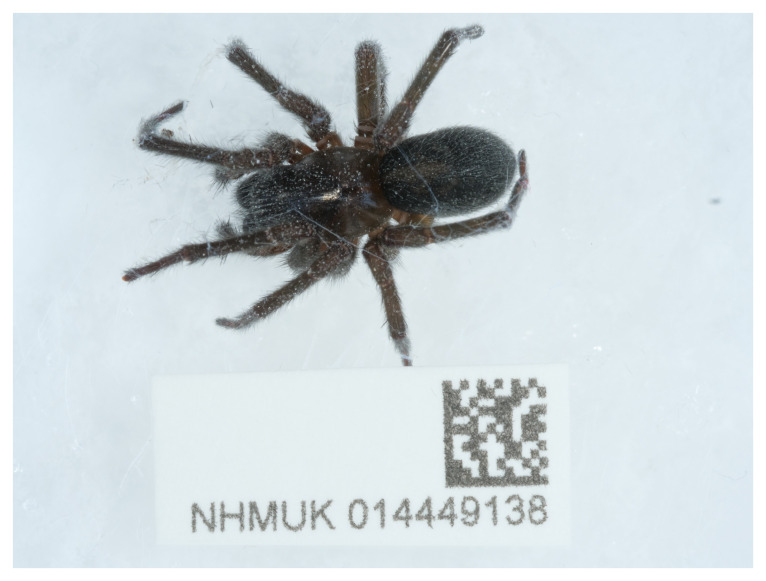
Photograph of the *Amaurobius ferox* (qqAmaFero1) specimen used for genome sequencing.

The genome was sequenced from one female *Amaurobius ferox* ( Figure 1) collected from Chobham Common, Otter Shaw, Surrey, UK (51.37, –0.59). A total of 31-fold coverage in Pacific Biosciences single-molecule HiFi long reads was generated. Primary assembly contigs were scaffolded with chromosome conformation Hi-C data. Manual assembly curation corrected 47 missing joins or mis-joins and removed 2 haplotypic duplications, reducing the scaffold number by 6.47%, and increasing the scaffold N50 by 1.35%.

**Figure 2.**
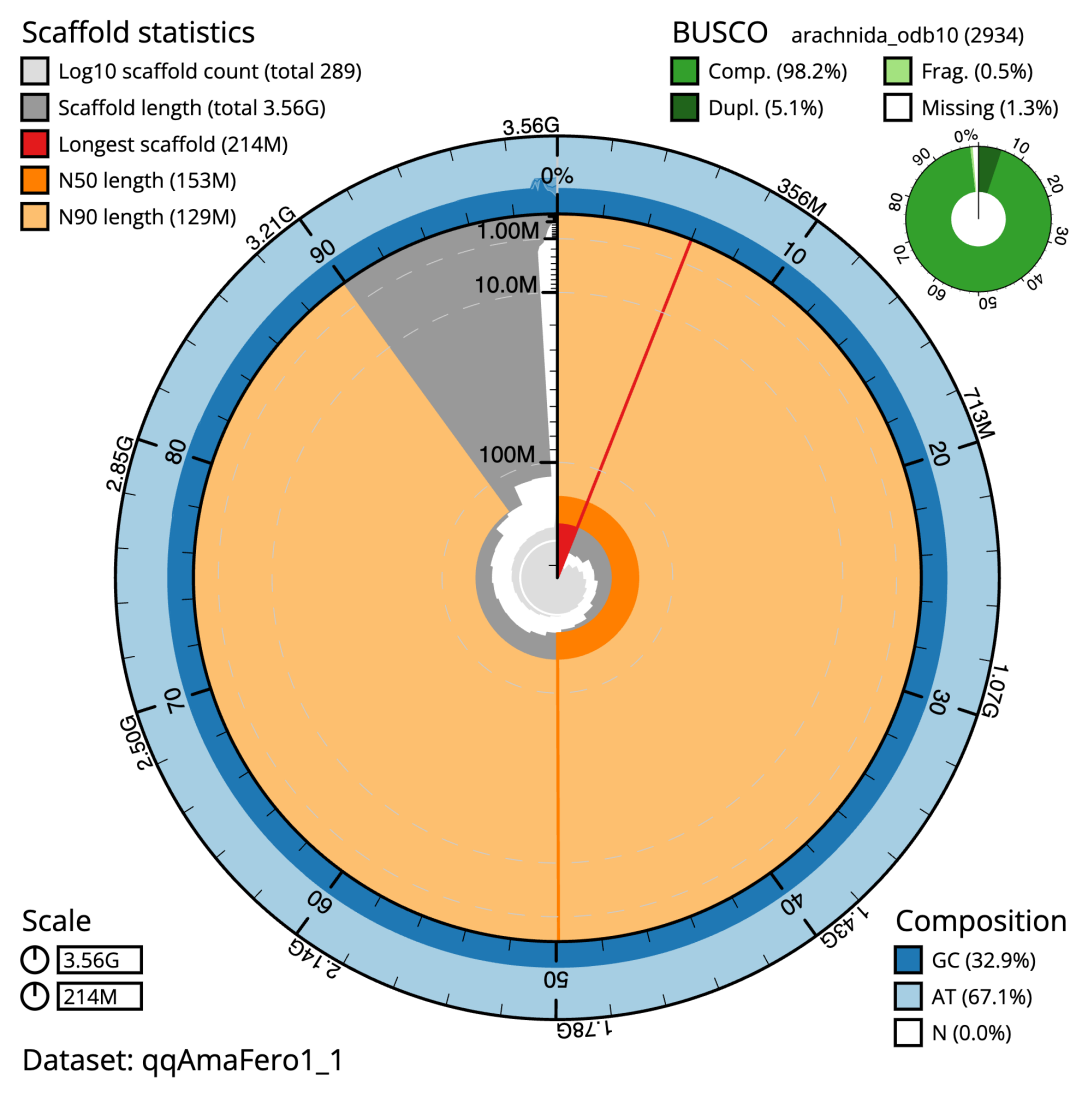
Genome assembly of *Amaurobius ferox*, qqAmaFero1.1: metrics. The BlobToolKit Snailplot shows N50 metrics and BUSCO gene completeness. The main plot is divided into 1,000 size-ordered bins around the circumference with each bin representing 0.1% of the 3,564,847,194 bp assembly. The distribution of scaffold lengths is shown in dark grey with the plot radius scaled to the longest scaffold present in the assembly (214,309,029 bp, shown in red). Orange and pale-orange arcs show the N50 and N90 scaffold lengths (153,399,618 and 128,711,411 bp), respectively. The pale grey spiral shows the cumulative scaffold count on a log scale with white scale lines showing successive orders of magnitude. The blue and pale-blue area around the outside of the plot shows the distribution of GC, AT and N percentages in the same bins as the inner plot. A summary of complete, fragmented, duplicated and missing BUSCO genes in the arachnida_odb10 set is shown in the top right. An interactive version of this figure is available at https://blobtoolkit.genomehubs.org/view/qqAmaFero1_1/dataset/qqAmaFero1_1/snail.

**Figure 3.**
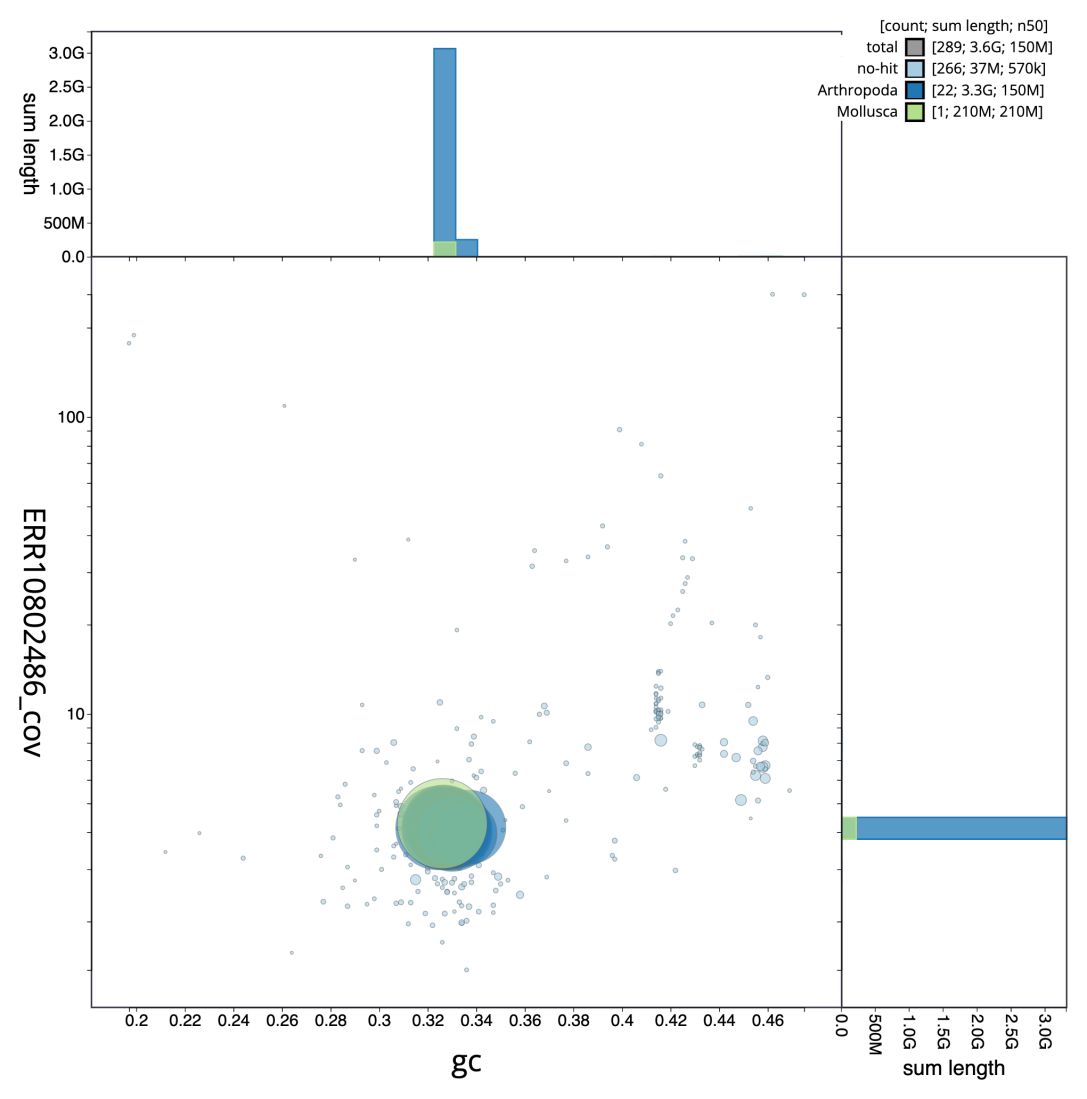
Genome assembly of *Amaurobius ferox*, qqAmaFero1.1: BlobToolKit GC-coverage plot. Scaffolds are coloured by phylum. Circles are sized in proportion to scaffold length. Histograms show the distribution of scaffold length sum along each axis. An interactive version of this figure is available at https://blobtoolkit.genomehubs.org/view/qqAmaFero1_1/dataset/qqAmaFero1_1/blob.

**Figure 4.**
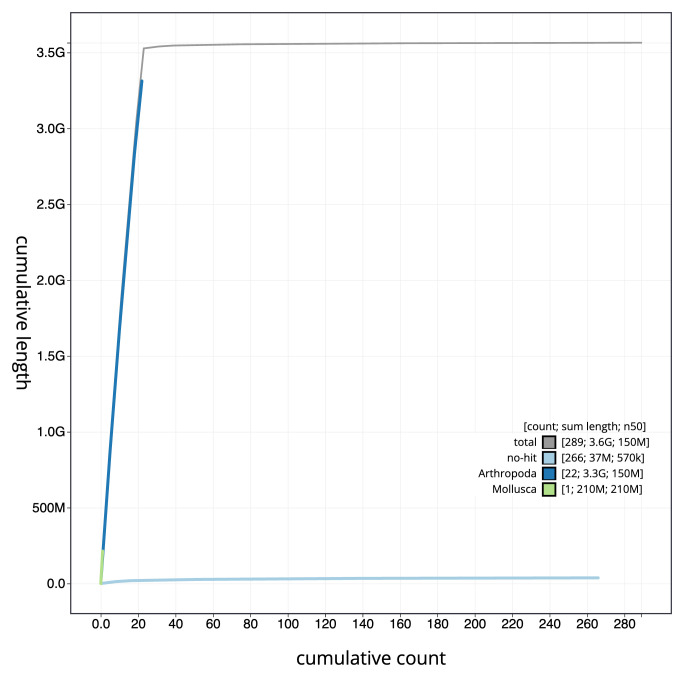
Genome assembly of *Amaurobius ferox*, qqAmaFero1.1: BlobToolKit cumulative sequence plot. The grey line shows cumulative length for all scaffolds. Coloured lines show cumulative lengths of scaffolds assigned to each phylum using the buscogenes taxrule. An interactive version of this figure is available at https://blobtoolkit.genomehubs.org/view/qqAmaFero1_1/dataset/qqAmaFero1_1/cumulative.

**Figure 5.**
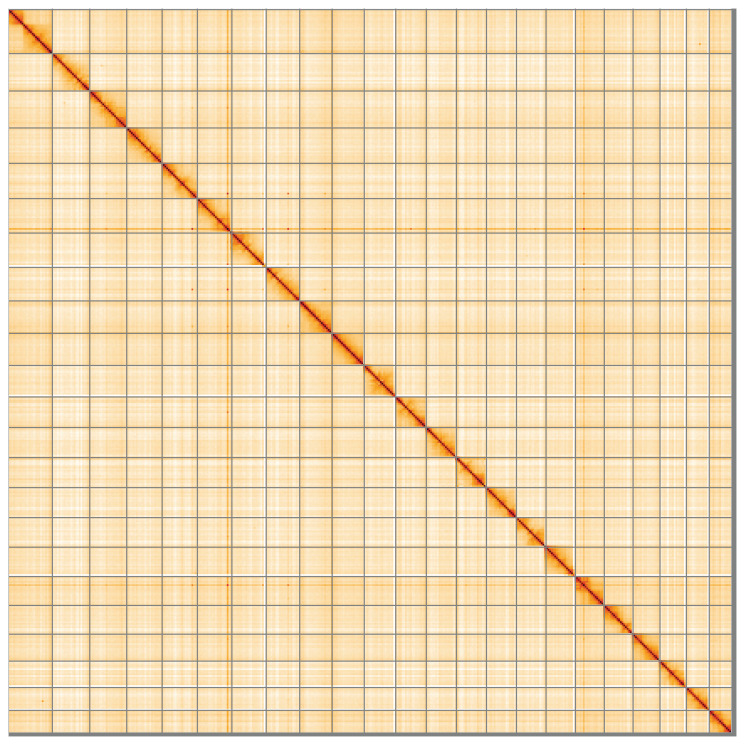
Genome assembly of *Amaurobius ferox*, qqAmaFero1.1: Hi-C contact map of the qqAmaFero1.1 assembly, visualised using HiGlass. Chromosomes are shown in order of size from left to right and top to bottom. An interactive version of this figure may be viewed at https://genome-note-higlass.tol.sanger.ac.uk/l/?d=dv9liBZBQZmqK02adfoALw.

The final assembly has a total length of 3564.8 Mb in 288 sequence scaffolds with a scaffold N50 of 153.4 Mb ( Table 1). The snailplot in Figure 2 provides a summary of the assembly statistics, while the distribution of assembly scaffolds on GC proportion and coverage is shown in Figure 3. The cumulative assembly plot in Figure 4 shows curves for subsets of scaffolds assigned to different phyla. Most (99%) of the assembly sequence was assigned to 23 chromosomal-level scaffolds, representing 20 autosomes and the X _1_, X _2_ and X _3_ sex chromosomes. Chromosome-scale scaffolds confirmed by the Hi-C data are named in order of size ( Figure 5; Table 2). The X chromosomes were assigned based on synteny to *Dolomedes plantarius* (GCA_907164885.2). While not fully phased, the assembly deposited is of one haplotype. Contigs corresponding to the second haplotype have also been deposited. The mitochondrial genome was also assembled and can be found as a contig within the multifasta file of the genome submission.

**Table 1.** Genome data for *Amaurobius ferox*, qqAmaFero1.1.

Project accession data		
Assembly identifier	qqAmaFero1.1	
Species	*Amaurobius ferox*	
Specimen	qqAmaFero1	
NCBI taxonomy ID	336583	
BioProject	PRJEB59161	
BioSample ID	SAMEA8534437	
Isolate information	qqAmaFero1 qqAmaFero1,qqAmaFero2 (Hi-C sequencing)	
Assembly metrics *		*Benchmark*
Consensus quality (QV)	61.4	*≥ 50*
*k*-mer completeness	100.0%	*≥ 95%*
BUSCO **	C:98.2%[S:93.0%,D:5.1%],F:0.5%,M:1.3%,n:2,934	*C ≥ 95%*
Percentage of assembly mapped to chromosomes	99%	*≥ 95%*
Sex chromosomes	X _1_, X _2_, X _3_	*localised homologous pairs*
Organelles	Mitochondrial genome: 14.24 kb	*complete single alleles*
Raw data accessions		
PacificBiosciences SEQUEL II	ERR10812849, ERR10809399, ERR10809400, ERR10809401, ERR10812848	
Hi-C Illumina	ERR10802489	
PolyA RNA-Seq Illumina	ERR11837464	
Genome assembly		
Assembly accession	GCA_951213105.1	
*Accession of alternate haplotype*	GCA_951215355.1	
Span (Mb)	3,564.8	
Number of contigs	1,796	
Contig N50 length (Mb)	4.4	
Number of scaffolds	288	
Scaffold N50 length (Mb)	153.4	
Longest scaffold (Mb)	214.31	

* Assembly metric benchmarks are adapted from column VGP-2020 of “Table 1: Proposed standards and metrics for defining genome assembly quality” from Rhie *et al.* (2021). ** BUSCO scores based on the arachnida_odb10 BUSCO set using version 5.3.2. C = complete [S = single copy, D = duplicated], F = fragmented, M = missing, n = number of orthologues in comparison. A full set of BUSCO scores is available at https://blobtoolkit.genomehubs.org/view/qqAmaFero1_1/dataset/qqAmaFero1_1/busco.

**Table 2.** Chromosomal pseudomolecules in the genome assembly of *Amaurobius ferox*, qqAmaFero1.

INSDC accession	Chromosome	Length (Mb)	GC%
OX578134.1	1	182.28	32.5
OX578135.1	2	180.23	33.0
OX578136.1	3	172.74	32.5
OX578137.1	4	171.82	33.0
OX578138.1	5	168.11	33.0
OX578139.1	6	167.27	33.0
OX578140.1	7	163.42	32.5
OX578143.1	8	153.4	33.0
OX578144.1	9	149.26	33.0
OX578145.1	10	146.64	33.0
OX578146.1	11	146.52	33.0
OX578147.1	12	146.11	33.0
OX578148.1	13	144.35	32.5
OX578149.1	14	143.16	33.0
OX578150.1	15	140.84	33.5
OX578151.1	16	140.23	33.0
OX578152.1	17	131.31	33.0
OX578153.1	18	128.71	33.0
OX578154.1	19	111.33	33.0
OX578155.1	20	110.67	33.5
OX578156.1	MT	0.01	20.0
OX578133.1	X _1_	214.31	32.5
OX578141.1	X _2_	158.19	32.5
OX578142.1	X _3_	156.49	32.5

The estimated Quality Value (QV) of the final assembly is 61.4 with *k*-mer completeness of 100.0%, and the assembly has a BUSCO v5.3.2 completeness of 98.2% (single = 93.0%, duplicated = 5.1%), using the arachnida_odb10 reference set ( *n* = 2,934).

Metadata for specimens, barcode results, spectra estimates, sequencing runs, contaminants and pre-curation assembly statistics are given at https://links.tol.sanger.ac.uk/species/336583.

## Methods

### Sample acquisition and nucleic acid extraction

The specimen used for genome sequencing was a female *Amaurobius ferox* (specimen ID NHMUK014449138, ToLID qqAmaFero1), which was collected from Chobham Common, Otter Shaw, Surrey, UK (latitude 51.37, longitude –0.59) on 2020-10-03. The specimens used for Hi-C sequencing, also a female, (specimen ID NHMUK014444547, ToLID qqAmaFero2) and for RNA sequencing, a male, (specimen ID NHMUK014444548, ToLID qAmaFero3) were collected on the same occasion. The specimens were collected and identified by Sergio Henriques (Zoological Society of London) and preserved in liquid nitrogen.

The workflow for high molecular weight (HMW) DNA extraction at the WSI includes a sequence of core procedures: sample preparation; sample homogenisation, DNA extraction, fragmentation, and clean-up. In sample preparation, the qqAmaFero1 sample was weighed and dissected on dry ice ( Jay *et al.*, 2023). Tissue from the cephalothorax was homogenised using a PowerMasher II tissue disruptor ( Denton *et al.*, 2023a). HMW DNA was extracted using the Automated MagAttract v1 protocol ( Sheerin *et al.*, 2023). DNA was sheared into an average fragment size of 12–20 kb in a Megaruptor 3 system with speed setting 30 ( Todorovic *et al.*, 2023). Sheared DNA was purified by solid-phase reversible immobilisation ( Strickland *et al.*, 2023): in brief, the method employs a 1.8X ratio of AMPure PB beads to sample to eliminate shorter fragments and concentrate the DNA. The concentration of the sheared and purified DNA was assessed using a Nanodrop spectrophotometer and Qubit Fluorometer and Qubit dsDNA High Sensitivity Assay kit. Fragment size distribution was evaluated by running the sample on the FemtoPulse system.

RNA was extracted from abdomen tissue of qqAmaFero3 in the Tree of Life Laboratory at the WSI using the RNA Extraction: Automated MagMax™ *mir*Vana protocol ( do Amaral *et al.*, 2023). The RNA concentration was assessed using a Nanodrop spectrophotometer and a Qubit Fluorometer using the Qubit RNA Broad-Range Assay kit. Analysis of the integrity of the RNA was done using the Agilent RNA 6000 Pico Kit and Eukaryotic Total RNA assay.

Protocols developed by the Tree of Life laboratory are publicly available on protocols.io ( Denton *et al.*, 2023b).

### Sequencing

Pacific Biosciences HiFi circular consensus DNA sequencing libraries were constructed according to the manufacturers’ instructions. Poly(A) RNA-Seq libraries were constructed using the NEB Ultra II RNA Library Prep kit. DNA and RNA sequencing was performed by the Scientific Operations core at the WSI on Pacific Biosciences SEQUEL II (HiFi) and Illumina NovaSeq 6000 (RNA-Seq) instruments. Hi-C data were also generated from cephalothorax tissue of qqAmaFero2 using the Arima2 kit and sequenced on the Illumina NovaSeq 6000 instrument.

### Genome assembly, curation and evaluation

Assembly was carried out with Hifiasm ( Cheng *et al.*, 2021) and haplotypic duplication was identified and removed with purge_dups ( Guan *et al.*, 2020). The assembly was then scaffolded with Hi-C data ( Rao *et al.*, 2014) using YaHS ( Zhou *et al.*, 2023). The assembly was checked for contamination and corrected using the gEVAL system ( Chow *et al.*, 2016) as described previously ( Howe *et al.*, 2021). Manual curation was performed using gEVAL, HiGlass ( Kerpedjiev *et al.*, 2018) and PretextView ( Harry, 2022). The mitochondrial genome was assembled using MitoHiFi ( Uliano-Silva *et al.*, 2023), which runs MitoFinder ( Allio *et al.*, 2020) or MITOS ( Bernt *et al.*, 2013) and uses these annotations to select the final mitochondrial contig and to ensure the general quality of the sequence.

A Hi-C map for the final assembly was produced using bwa-mem2 ( Vasimuddin *et al.*, 2019) in the Cooler file format ( Abdennur & Mirny, 2020). To assess the assembly metrics, the *k*-mer completeness and QV consensus quality values were calculated in Merqury ( Rhie *et al.*, 2020). This work was done using Nextflow ( Di Tommaso *et al.*, 2017) DSL2 pipelines “sanger-tol/readmapping” ( Surana *et al.*, 2023a) and “sanger-tol/genomenote” ( Surana *et al.*, 2023b). The genome was analysed within the BlobToolKit environment ( Challis *et al.*, 2020) and BUSCO scores ( Manni *et al.*, 2021; Simão *et al.*, 2015) were calculated.

Table 3 contains a list of relevant software tool versions and sources.

**Table 3.** Software tools: versions and sources.

Software tool	Version	Source
BlobToolKit	4.2.1	https://github.com/blobtoolkit/blobtoolkit
BUSCO	5.3.2	https://gitlab.com/ezlab/busco
gEVAL	N/A	https://geval.org.uk/
Hifiasm	0.16.1-r375	https://github.com/chhylp123/hifiasm
HiGlass	1.11.6	https://github.com/higlass/higlass
Merqury	MerquryFK	https://github.com/thegenemyers/MERQURY.FK
MitoHiFi	3	https://github.com/marcelauliano/MitoHiFi
PretextView	0.2	https://github.com/wtsi-hpag/PretextView
purge_dups	1.2.3	https://github.com/dfguan/purge_dups
sanger-tol/genomenote	v1.0	https://github.com/sanger-tol/genomenote
sanger-tol/readmapping	1.1.0	https://github.com/sanger-tol/readmapping/tree/1.1.0
YaHS	1.2a	https://github.com/c-zhou/yahs

### Wellcome Sanger Institute – Legal and Governance

The materials that have contributed to this genome note have been supplied by a Darwin Tree of Life Partner. The submission of materials by a Darwin Tree of Life Partner is subject to the **‘Darwin Tree of Life Project Sampling Code of Practice’**, which can be found in full on the Darwin Tree of Life website here. By agreeing with and signing up to the Sampling Code of Practice, the Darwin Tree of Life Partner agrees they will meet the legal and ethical requirements and standards set out within this document in respect of all samples acquired for, and supplied to, the Darwin Tree of Life Project. 

Further, the Wellcome Sanger Institute employs a process whereby due diligence is carried out proportionate to the nature of the materials themselves, and the circumstances under which they have been/are to be collected and provided for use. The purpose of this is to address and mitigate any potential legal and/or ethical implications of receipt and use of the materials as part of the research project, and to ensure that in doing so we align with best practice wherever possible. The overarching areas of consideration are:

• Ethical review of provenance and sourcing of the material

• Legality of collection, transfer and use (national and international) 

Each transfer of samples is further undertaken according to a Research Collaboration Agreement or Material Transfer Agreement entered into by the Darwin Tree of Life Partner, Genome Research Limited (operating as the Wellcome Sanger Institute), and in some circumstances other Darwin Tree of Life collaborators.

## Data Availability

European Nucleotide Archive: *Amaurobius ferox*. Accession number PRJEB59161; https://identifiers.org/ena.embl/PRJEB59161 ( Wellcome Sanger Institute, 2023). The genome sequence is released openly for reuse. The *Amaurobius ferox* genome sequencing initiative is part of the Darwin Tree of Life (DToL) project. All raw sequence data and the assembly have been deposited in INSDC databases. The genome will be annotated using available RNA-Seq data and presented through the Ensembl pipeline at the European Bioinformatics Institute. Raw data and assembly accession identifiers are reported in Table 1.

## Author information

Members of the Natural History Museum Genome Acquisition Lab are listed here: https://doi.org/10.5281/zenodo.7139035.

Members of the Darwin Tree of Life Barcoding collective are listed here: https://doi.org/10.5281/zenodo.4893703.

Members of the Wellcome Sanger Institute Tree of Life Management, Samples and Laboratory team are listed here: https://doi.org/10.5281/zenodo.10066175.

Members of Wellcome Sanger Institute Scientific Operations: Sequencing Operations are listed here: https://doi.org/10.5281/zenodo.10043364.

Members of the Wellcome Sanger Institute Tree of Life Core Informatics team are listed here: https://doi.org/10.5281/zenodo.10066637.

Members of the Tree of Life Core Informatics collective are listed here: https://doi.org/10.5281/zenodo.5013541.

Members of the Darwin Tree of Life Consortium are listed here: https://doi.org/10.5281/zenodo.4783558.
