# Supplementary material for: The genome sequence of the Black Lace-weaver spider, Amaurobius ferox (Walckenaer, 1830)
Source: Wellcome Open Res. 2024 Mar 1;9:105. [Version 1] doi: 10.12688/wellcomeopenres.21080.1 (PMC11249506; doi:10.12688/wellcomeopenres.21080.1)
Supplement: Peer review history

# Reviewer report: Reviewer response for version 1

**DOI:** 10.21956/wellcomeopenres.23319.r88302

**Published:** 2024-08-12

**Authors:** Natascha Zhang, Chetan Munegowda

**Affiliations:** 1 Department of Biology II - Evolutionary Ecology, Ludwig-Maximilians-University of Munich, Munich, Germany; 2 Justus-Liebig-Universitat Giessen Fachbereich 08 Biologie und Chemie, Giessen, Hesse, Germany

The manuscript with the title “The genome sequence of the Black Lace-weaver spider, *Amaurobius ferox*” summarises general findings of a high-quality genome assembly of the nocturnal spider *Amaurobius ferox* as part of the DToL project.

 There is no doubt that the quality of the genome and the assembly as well as the figures are convincingly of high quality. The authors sufficiently describe sample collection, software used. Although being part of the DToL, the manuscript would benefit a bit more from highlighting the choice of the species, the initial introduction is already there but it would be nice to known if this is the first chromosome level genome of a spider practicing matriphagy and what effect this complex way of maternal care might have on selection and by that genome evolution. In addition, the results presented in the paper could be more described a bit more thoroughly also in the main text, just naming a few of the assembly statistics etc. and provide some comparisons and conclusion to other genomes.

 In the methods I would recommend giving some rationale why three different individuals were used for the sequencing, and if it was checked to cause any problems with the assembly, even if it was an obvious reason like the size of the individual, I would still discuss this drawback. Especially the fact that females were used for the genome sequencing but a male for the RNA sequencing.

 Another major issue which was already highlighted by another reviewer is that although each version and software used is indicated, the parameters are not given. As well as the sequencing depth is missing.

Are sufficient details of methods and materials provided to allow replication by others?

No

Is the rationale for creating the dataset(s) clearly described?

No

Are the datasets clearly presented in a useable and accessible format?

Yes

Are the protocols appropriate and is the work technically sound?

Yes

Reviewer Expertise:

developmental biology, RNAseq, Evo Devo, genomics

We confirm that we have read this submission and believe that we have an appropriate level of expertise to confirm that it is of an acceptable scientific standard, however we have significant reservations, as outlined above.

# Reviewer report: Reviewer response for version 1

**DOI:** 10.21956/wellcomeopenres.23319.r86923

**Published:** 2024-07-15

**Authors:** Zhi-Sheng Zhang

**Affiliations:** 1 Southwest University, Chongqing, China

This paper presents a genome assembly from an individual female *Amaurobius ferox*.

 The genome sequence is 3564.8 megabases in span and is scaffolded into 23 chromosomal pseudomolecules, including the X1, X2 and X3 sex chromosome. The rationale for creating the dataset(s)is clearly described. The protocols are appropriate, and the works are technically sound, there are sufficient details of methods and materials provided to allow replication by others.

Are sufficient details of methods and materials provided to allow replication by others?

Yes

Is the rationale for creating the dataset(s) clearly described?

Yes

Are the datasets clearly presented in a useable and accessible format?

Yes

Are the protocols appropriate and is the work technically sound?

Yes

Reviewer Expertise:

plylogenomics

I confirm that I have read this submission and believe that I have an appropriate level of expertise to confirm that it is of an acceptable scientific standard.

# Reviewer report: Reviewer response for version 1

**DOI:** 10.21956/wellcomeopenres.23319.r88303

**Published:** 2024-07-01

**Authors:** Madeleine Aase-Remedios

**Affiliations:** 1 Department of Biosciences, Durham University, Durham, UK

The authors present a high quality genome assembly for the lace spider *Amaurobius ferox* for public use, using state of the art methods. While rationale is not explicitly given, it is obviously part of the larger DToL project's aims.

 RNA was sequenced, but the annotation is not yet included. I would prefer annotations to be published with assemblies, but I understand the benefit of making the genome public asap for use by other researchers.

 The methods does not include many details to make this fully replicable (on parameters used, etc.). The authors provide links to their standard pipeline, and various protocols, so technically it's fine, just not ideal.

 The background provides a concise summary of the habitat and identification of the species. Overall, the content is sufficient for a data note.

Are sufficient details of methods and materials provided to allow replication by others?

Yes

Is the rationale for creating the dataset(s) clearly described?

Yes

Are the datasets clearly presented in a useable and accessible format?

Yes

Are the protocols appropriate and is the work technically sound?

Yes

Reviewer Expertise:

Arachnid development, genomics, and transcriptomics

I confirm that I have read this submission and believe that I have an appropriate level of expertise to confirm that it is of an acceptable scientific standard.
